# A Role for Endoplasmic Reticulum Stress in Intracerebral Hemorrhage

**DOI:** 10.3390/cells9030750

**Published:** 2020-03-19

**Authors:** Shaik Ismail Mohammed Thangameeran, Sheng-Tzung Tsai, Hsiang-Yi Hung, Wei-Fen Hu, Cheng-Yoong Pang, Shin-Yuan Chen, Hock-Kean Liew

**Affiliations:** 1 Institute of Medical Sciences, Tzu Chi University, Hualien 970, Taiwan; 106324122@gms.tcu.edu.tw (S.I.M.T.); flydream.tsai@gmail.com (S.-T.T.); cypang@gms.tcu.edu.tw (C.-Y.P.); william.sychen@msa.hinet.net (S.-Y.C.); 2 Department of Neurosurgery, Hualien Tzu Chi Hospital, Buddhist Tzu Chi Medical Foundation, Hualien 970, Taiwan; shura0714@yahoo.com.tw; 3 Neuro-Medical Scientific Center, Hualien Tzu Chi Hospital, Buddhist Tzu Chi Medical Foundation, Hualien 970, Taiwan; 4 PhD Program in Pharmacology and Toxicology, Tzu Chi University, Hualien 970, Taiwan; 103327105@gms.tcu.edu.tw; 5 Department of Medical Research, Hualien Tzu Chi Hospital, Buddhist Tzu Chi Medical Foundation, Hualien 970, Taiwan; 6 CardioVascular Research Center, Hualien Tzu Chi Hospital, Buddhist Tzu Chi Medical Foundation, Hualien 970, Taiwan

**Keywords:** intracerebral hemorrhage, ER stress, unfolded protein response, neuroinflammation, necroptosis, ferroptosis, pyroptosis

## Abstract

The endoplasmic reticulum (ER) is an intracellular organelle that performs multiple functions, such as lipid biosynthesis, protein folding, and maintaining intracellular calcium homeostasis. Thus, conditions wherein the ER is unable to fold proteins is defined as ER stress, and an inbuilt quality control mechanism, called the unfolded protein response (UPR), is activated during ER stress, which serves as a recovery system that inhibits protein synthesis. Further, based on the severity of ER stress, the response could involve both proapoptotic and antiapoptotic phases. Intracerebral hemorrhage (ICH) is the second most common subtype of cerebral stroke and many lines of evidence have suggested a role for the ER in major neurological disorders. The injury mechanism during ICH includes hematoma formation, which in turn leads to inflammation, elevated intracranial pressure, and edema. A proper understanding of the injury mechanism(s) is required to effectively treat ICH and closing the gap between our current understanding of ER stress mechanisms and ICH injury can lead to valuable advances in the clinical management of ICH.

## 1. Introduction

Intracerebral hemorrhage (ICH) is a subtype of hemorrhagic stroke [1], morbidity, and mortality due to ICH is accounted depending on its location in the brain. Among all stroke subtypes, the proportion of ICH is different in various parts of the world; while in the USA, Europe, and Australia it accounts for only 10–15% of all strokes, surprisingly, this number can be as high as 20–30% among Asian populations [2]. ICH is also the second most common type of stroke in Taiwan, accounting for 16.1% of all strokes [3]. There are two stages of ICH-elicited injury, namely, primary brain injury (PBI) and secondary brain injury (SBI). PBI occurs due to the rupture of cerebral blood vessels predominantly because of cardiovascular conditions, with hypertension playing a major role. PBI is characterized by mechanical injury followed by a mass effect with the initial ictus causing physical tissue disruption that then leads to pathophysiological conditions in the brain.

**Figure 1:**
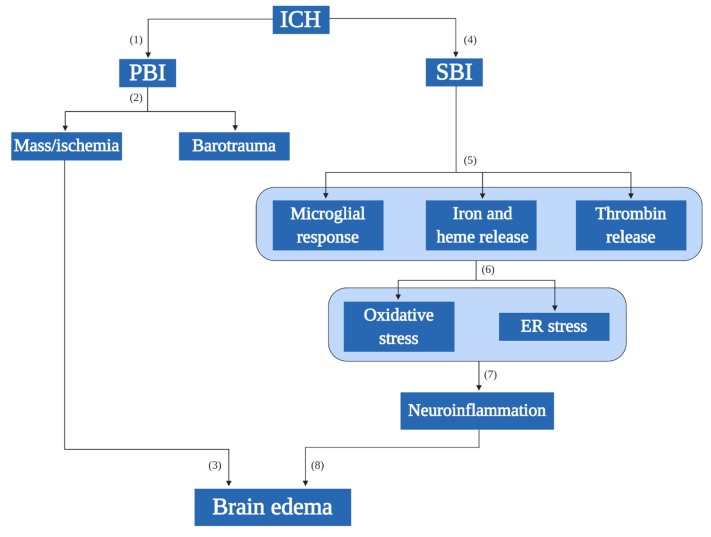
Pathology of intracerebral hemorrhage (ICH). Primary brain injury (PBI; 1 is the initial ictus that happens due to physical damage (2), which in turn leads to the mass effect or the ischemic effect that increases the volume of the hematoma (barotrauma). The mass/ischemic effect can directly influence the increase of the brain edema (3). The pathophysiology of ICH involves other series of steps called (4) the secondary brain injury (SBI, which is the long-term sequential aftereffects following ICH. The SBI events include (5) the release of thrombin, which acts as the initial response to repair the damaged site following which the host microglia come into play to engulf the erythrocytes. Since the initial ictus causes a clot in the site, there is an excessive release of clot components; majorly free iron and heme. (6) The course of events after SBI initiates the oxidative stress and ER stress consecutively contributing to (7) the neuroinflammation. (8) The progression of SBI events eventually leads to a brain edema. ICH: intracerebral hemorrhage, PBI: primary brain injury, SBI: secondary brain injury.

A sudden rise in intracranial hematoma volume causes an increase in barotrauma and reduces blood flow to the area of the ictus [4,5]. Typically, PBI is followed by SBI, which is considered as a devastating stage after ICH, and the severity of SBI depends on the rate of recovery and location of the ICH. SBI involves the neuroinflammatory response to the triggering of the coagulation cascade through activation of the immune system. The inflammation size is based on the volume and position of the hematoma [5,6,7]. The various pathological factors responsible for SBI include the host immune response, release of thrombin, release of clot components (iron and heme), increased cytotoxicity, and inflammation [5,7]. The initial response to an ICH is the disruption of the blood–brain barrier, which causes the coagulation components to rush to the bleeding side, which involves the activation of microglia (the primary immune response cells; Figure 1) [8]. Activated microglia secrete numerous proinflammatory cytokines such as the tumor necrosis factor (TNF)-α, interleukin (IL)-6, and IL-1β that ultimately lead to neuroinflammation [9], and these proinflammatory cytokines, when present at the injury site, tend to cause inflammation in situ [10]. Additionally, immediate lysis of red blood cells (RBCs) after ICH promotes the release of free hemoglobin, which is potentially neurotoxic, and it is degraded to heme and iron by an enzyme called heme oxygenase-1 (HO-1). Iron and heme toxicity play a vital role in SBI [11,12]. All these aforementioned pathophysiological stages, namely microglial activation, heme and iron release, can generate free radicals such as reactive oxygen species (ROS) and reactive nitrogen species, which can mediate neuroinflammation [6]. While the various mechanisms involved in neuroinflammation after ICH and associated modes of cell death are topics of interest, we have focused this review on understanding the role of ER stress in various cell death mechanisms after the initial ictus.

## 2. General Therapeutic Interventions for ICH

The initial screening for ICH uses imaging tools such as CT or MRI and this typically takes 3 days. This basic screening will also provide information on the location and volume of ICH, and at the end of the first phase, physicians will be able to differentiate among ICH, intraventricular hemorrhage, subarachnoid hemorrhage (SAH), ischemic stroke, primary brain tumors, and metastasis [13]. However, the subsequent procedure is CT angiography spot signs, even though this procedure helps identify active hemorrhage and understand the rate of ongoing bleeding its use is still under debate. Despite obvious advantages, the usage of CT angiography spot signs is not standard practice yet [14,15]. Following the first and second phase of diagnostics, the ICH score is evaluated to improve standard of the clinical management. This process involves assessing the Glasgow coma scale and other parameters that are necessary to arrive at an appropriate clinical management strategy [16].

**Figure 2:**
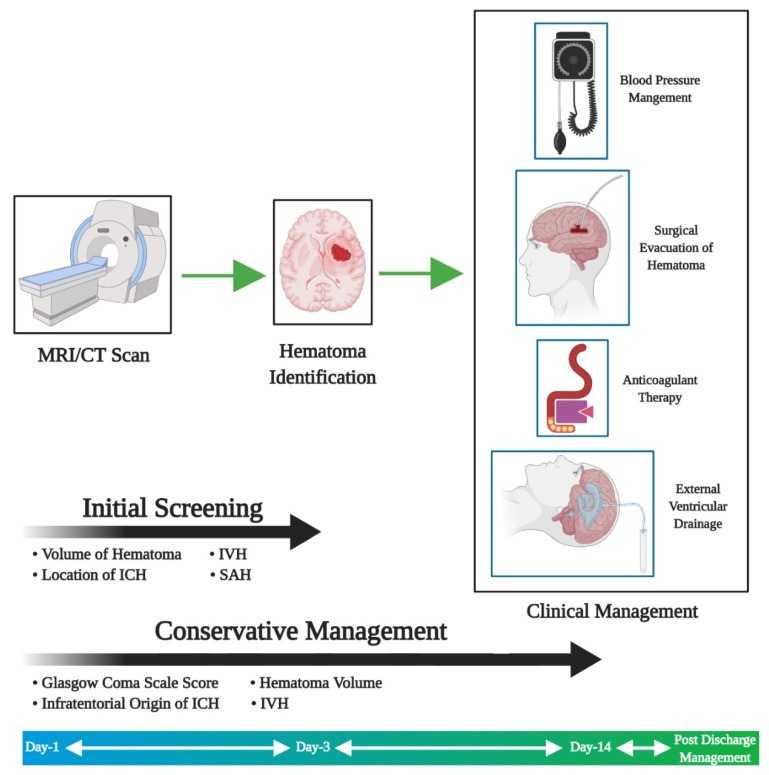
Clinical management of ICH. The steps involved in the clinical management of ICH are initial screening to analyze the clinical conditions followed by the screening procedure to understand the treatment needs of the patient. Based on the first three processes the clinical management is decided between surgical procedures or conservative management.

Major factors taken into account during treatment strategy planning are age, neurological deficits, and blood pressure. Clinical management can include medical treatment for lowering blood pressure, coagulopathy, or even surgery (e.g., external ventricular drainage or hematoma evacuation; Figure 2) [17].

## 3. Cell Death Pathways after ICH

### 3.1. Necrosis, Apoptosis, and Necroptosis

Cell death after ICH is predominantly necrosis due to cellular disintegration; however, evidence of apoptosis has been documented in the form of DNA fragmentation using electrophoresis [18]. Additionally, a retrospective clinical study has suggested that apoptosis might be a major mechanism of cell death in ICH among human subjects in the perihematomal region [19]. Some studies have also investigated if a combination of both apoptosis and necrosis occurs after ICH in rat and rabbit models [20,21]. Necroptosis is programmed necrosis that occurs upon stimulation by proinflammatory cytokines, especially TNF-α. The term ‘Necroptosis’ refers to a non-apoptotic cell death pathway and was coined by a study that focused on the role of a drug named necrostatin-1 (nec-1), which blocks the genetic determinant receptor-interacting serine/threonine-protein kinase 1 (RIPK1). Necroptosis induces inflammation, thereby slowing down the recovery process, and RIPK1 is responsible for inducing necroptosis in the cells treated with TNF-α [22]. Recent insights into the therapeutic efficacy of nec-1 after ICH include its ability to inhibit TNF-α -induced necroptosis to limit cell death and a reduction in hematoma volume in mice [23,24]. Interestingly, one study showed that either ferroptosis or necroptosis alone can sufficiently drive cells towards cell death pathways after ICH and that inhibition of either of these pathways can steer the neuronal cell towards survival [25]. While other unconventional cell death pathways like ferroptosis and pyroptosis have also been reported as possible mechanisms of ICH-induced cell death.

### 3.2. Autophagy

The exact mode of cell death after ICH remains unresolved; nonetheless, the three major morphologically distinct cell death pathways of necrosis, apoptosis, and autophagy are common in ICH. The formation of autophagosomes requires the conversion of light chain-3 (LC3-I), which is cytosolic under normal conditions, to membrane bound LC3-II, and this conversion of LC3-1 to LC3-II is seen in ICH-induced rats. The existence of autophagy was determined by the morphology of the cells using electron microscopy and ICH rats showed an increased number of autophagic vacuoles in the perihematomal area, confirming a role for autophagy in ICH-induced cell death. Furthermore, an iron chelator inhibited the LC3-I to LC3-II conversion, underscoring the importance of iron in ICH-induced autophagy in rats which underwent autologous blood infusion ICH surgery [26].

### 3.3. Pyroptosis

Pyroptosis has been recently gaining greater research attention as an important cell death pathway. It is different from apoptotic and autophagic cell death in that it requires inflammasomes and is mediated by caspase-1, i.e., it is a cysteine aspartate protease-dependent cell death pathway. The most common inflammasome sensors are absent in melanoma 2 (AIM2), and pyrin and nucleotide-binding oligomerization domain-like receptors (NLRs). The most prevalently studied inflammasome is NLR pyrin domain containing 3 (NLRP3) and it is thought to mediate various neurodegenerative and neurological disorders [22,27]. Pyroptosis involves the release of proinflammatory cytokines, especially IL-1β/IL-18; this, when combined with caspase-1 and inflammasome activity, causes the cells to swell and burst, thus leading to cell death. Simultaneously, the released proinflammatory cytokines attract other immune cells, causing excessive inflammation. [28]. Caspase-1-dependent cell death has been documented after ICH, along with a role for P2X7 receptor-mediated pyroptotic cell death in hemorrhagic stroke [29]. Importantly, inhibition of caspase-1 by AC-YVAD-CMK (a selective inhibitor of caspase-1) reduced brain injury in a collagenase-induced mice model of ICH [29,30].

### 3.4. Ferroptosis

**Figure 3:**
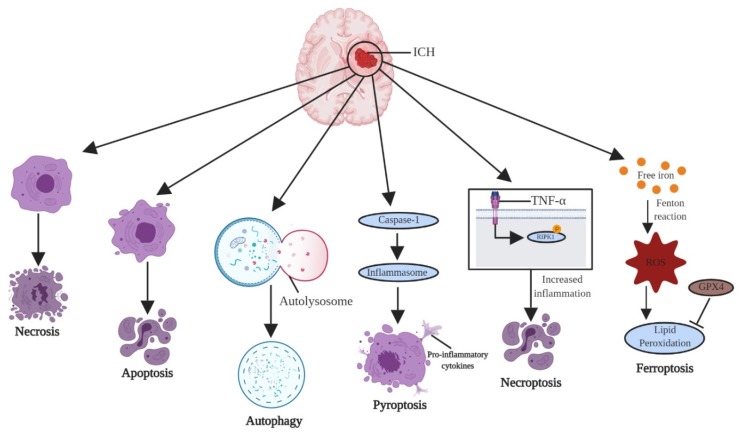
Cell death pathways in ICH. ICH includes all the three conventional forms of cell death (necrosis, apoptosis, and autophagy). Apart from regular cell death pathway, ICH also involves pyroptosis, necroptosis, and ferroptosis.

Ferroptosis is an iron-dependent form of non-apoptotic cell death. Activated microglia and infiltrating macrophages at the injury site engulf hemoglobin, degrade it, and release ferrous iron, which drives the production of ROS and results in lipid peroxidation; lipid peroxidation occurs when glutathione peroxidase 4 (GPX4) activity is inhibited. As there is excessive hemoglobin release after cell lysis in the post-ICH phase, the microglia accumulate surplus ferrous iron, which is transferred out of the microglia and accumulates in the neurons. This ferrous iron acts as the catalyst forming hydroxyl radicals through the Fenton reaction, and both hydroxyl radical and hydrogen peroxide are lethal ROS that elicit a neuroinflammatory response. The excessive release of hemoglobin after ICH due to erythrocyte lysis further increases ferroptosis post-ICH. A role for ferroptosis in ICH was demonstrated by inhibiting it using a specific inhibitor, ferrostatin-1, to rescue neuronal cell death due to excessive iron deposition. Interestingly ferrostatin-1 was able to reduce neuronal death by inhibiting lipid ROS and cyclooxygenase (COX)-2 expression [31,32]. Figure 3 provides the illustrated form of cell death pathways after ICH.

## 4. Endoplasmic Reticulum Stress

**Figure 4:**
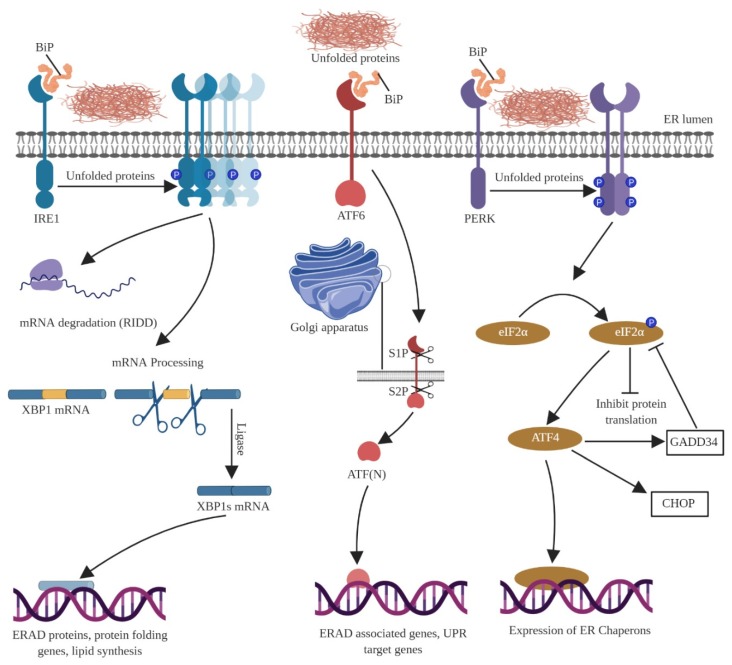
The molecular pathways of the unfolded protein response (UPR. During ER stress the three ER transducers (IRE1, PERK, and ATF6) tend to get activated and start the UPR mechanism to counteract the ER stress and to bring back the homeostasis. Failure to negate the ER stress will result in cell death. IRE1 undergoes oligomerization in response to unfolded proteins and the BiP gets dissociated from IRE1. IRE1 undergoes either mRNA degradation or mRNA processing with the help of XBP-1, which triggers the production of protein folding genes thereby mitigating ER stress. On the other hand, ATF6 gets into the secretory pathway inside the Golgi apparatus wherein the S1P and S2P cleave the ATF6. ATF6 fragments then translocate into the nucleus, which act as a transcription factor to generate UPR target genes. PERK first phosphorylates itself and also phosphorylates eIF2α thereby inactivating eIF2B, which further inhibits the translation of serine 51. This process leads to the translation of ATF4 producing ER chaperones.

ER is an integral organelle found in all eukaryotic cells and its vital functions include ensuring uninterrupted protein folding, synthesis of unsaturated fatty acids, and maintenance of intracellular Ca^2+^ homeostasis. Protein synthesis requires integration of the ER with the ribosome [33], and chaperones come into play to complete protein synthesis [34]. Various factors can perturb the normal function of the ER and cause ER stress, and these factors include hypoxia, hypoglycemia, hyperthermia, and improper calcium homeostasis [35]. Under stress conditions, the ER encounters aggregation of misfolded and unfolded proteins, which then elicits the UPR. The primary role of UPR is to restore normal function of the ER. Notably, cell death ensues if ER function cannot be restored, and the severity of ER stress determines whether cell death occurs through caspase-dependent or caspase-independent pathways [36]. The ER stress signal is primarily initiated by three effector proteins, the protein kinase RNA-like endoplasmic reticulum kinase (PERK), inositol-requiring protein-1 (IRE1), and activating transcription factor-6 (ATF6). These three transmembrane protein sensors are responsible for restoring homeostasis during UPR and are held in an inactive state by the binding immunoglobulin protein (BiP), also known as glucose regulated protein 78 (GRP78), which is a member of the heat shock protein-70 (hsp70) family. Normally, BiP is bound to the three effector proteins, but during ER stress or UPR, BiP disassociates from the effector proteins, which leads to sequential activation of downstream effector proteins. Illustrated form of UPR molecular pathways is given in Figure 4.

The two main functions of the three effector proteins in their active state are to cease protein synthesis to reduce ER stress and to increase the transcriptional activation of genes responsible for efficient protein folding [37]. The critical role(s) played by cell death signaling pathways involving ER transducers during the UPR has been a major area of research in the fields of neurodegenerative diseases, diabetes, and heart, kidney, and liver diseases [38]. Understanding all components of the UPR will provide greater insight about its activation or deactivation, which might be beneficial in the treatment of ICH. Thus, the arguments in favor of connecting the dots to understand the exact function of ER stress in inducing cell death post-ICH are compelling.

## 5. Molecular Pathways of ER Stress

### 5.1. ER Transducer—Inositol-Requiring Protein-1 (IRE1)

IRE1 is a type-1 ER transmembrane protein with an inner N-terminal luminal domain, a cytoplasmic domain, and an RNase domain. Normally, chaperones such as BiP bind to and prevent activation of IRE1; however, during ER stress, BiP disassociates from IRE1 and actively participates in the UPR. During UPR, the luminal domain of the IRE1 dissociates, which induces transautophosphorylation and conformational change of IRE1 that in turn activates its RNase domain. Activated IRE1 involves in the unconditional splicing of the mRNA of the transcription factor X-box binding protein-1 (XBP1), which further promotes the XBP1 dependent gene expression program. There are studies in yeast and mammalian cells that show the direct association of unfolded proteins with IRE1. The unfolded proteins directly bind with the luminal domain of IRE1. This association between unfolded proteins and IRE1 causes conformational change and oligomerization of IRE1 [39,40,41,42,43,44,45]. Data from *Caenorhabditis elegans* and mouse models demonstrate that, during UPR, IRE1-dependent downstream signaling is activated by splicing of mRNA that encodes XBP1 [46]. IRE1 is part of an inherent mechanism known as the regulated IRE1-dependent decay (RIDD), which has diverse effects on the cell that can lead to either preservation of homeostasis or cell death [47,48].

### 5.2. ER Transducer—Activating Transcription Factor 6 (ATF6)

As the name suggests, ATF6 is a transcription factor of the leucine zipper family that is localized to the ER and has a molecular weight of 50 kDa in its activated form. During ER stress, BiP dissociates from ATF6, which results in the exposure of its Golgi localization sequence [49]; ATF6 is then processed by Site-I (S1P) and Site-II (S2P) proteases followed by the release of ATF6 fragments [50]. These released ATF6 fragments enter the nucleus and induce promoters of the grp genes by activating the ER-stress-response elements [51]. Mammals exhibit two homologous ATF6 proteins, namely ATF6α and ATF6β [52], and grp genes are regulated by AFT6α after it enters the nucleus during ER stress. The functional importance of ATF6β remains less understood. ATF6α also plays a major role in inducing the nuclear expression of chaperones BiP and Xbp1 [53]. ATF6α-assisted induction of UPR mediators and chaperones is considered to be the prime switch that downregulates IRE1 signaling [54].

### 5.3. ER Transducer—Protein Kinase R-Like Endoplasmic Reticulum Kinase (PERK)

PERK is a type-I transmembrane protein, and as its translational role was first established using pancreatic cells, it is called pancreatic ER kinase or protein kinase RNA-like ER kinase [55]. PERK shares an identical domain assembly with IRE1 [56] and it is an ER-resident transmembrane kinase. The UPR activation is a mechanism to restore homeostasis through promoting protein folding via chaperones, degrading misfolded proteins, or slowing translation. This reduces the load of unfolded proteins and increases the efficiency of protein folding. While IRE1 and ATF6 activate genes responsible for mitigating protein folding capacity [57], unfolded protein load is controlled by PERK. The absence of PERK leads to excessive protein synthesis, which ultimately results in extreme ER stress and disruption of cell homeostasis ultimately resulting in cell death [58]. Under normal conditions, BiP is found attached to the luminal domain of the PERK protein; however, during ER stress, BiP disassociates from the luminal domain and helps lessen the increasing protein load [56]. Like IRE1, PERK also has a direct relationship between misfolded proteins and its oligomerization, which triggers the UPR [59]. PERK phosphorylates eukaryotic translation initiation factor 2α (eIF2α) on serine 51 and this phosphorylation inhibits eIF2B, ensuring the translation of ATF4. The translation of ATF-4 induces the CHOP genes and the growth arrest and DNA damage-inducible 34 (GADD34) genes. The former acts as a transcription factor that is responsible for apoptosis and the latter is a negative regulator that stops the UPR by dephosphorylating eIF2α with the help of protein phosphatase 1 (PP1c), thereby restarting the protein synthesis process [60,61]. 

### 5.4. Calcium Homeostasis and ER Stress

Apart from protein and lipid biosynthesis, the ER also serves as an essential Ca^2+^ storage site in eukaryotic cells. Ca^2+^ homeostasis is necessary for normal functioning of the cell and three main processes contribute to maintaining Ca^2+^ equilibrium in the ER. These are (i) ensuring that the Ca^2+^ store within the ER lumen is replenished from the cytosol; (ii) maintaining Ca^2+^ within the ER using binding proteins; and (iii) controlled release of calcium from the ER to the cytosol [62]. Thus, ER Ca^2+^ equilibrium is maintained by controlling the influx and the outflow of Ca^2+^. The main Ca^2+^ release machinery is regulated by ryanodine-receptor (RyR) and inositol 1,4,5-trisphosphate (IP3) receptor (IP3R) [63,64]. Upon binding to specific ligands (Ca^2+^ for RyR and IP3 for IP3R), RyR and IP3R tend to release Ca^2+^ from the ER, which reduces Ca^2+^ concentration within the ER [65]. This process is followed by replenishment of ER Ca^2+^ from extracellular sources through the plasma membrane; this is executed by store operated Ca^2+^ entry (SOCE) through calcium release-activated calcium channels. SOCE is modulated by the ER membrane protein stromal interaction molecule 1 and 2 (STIM1/2) and plasma membrane protein calcium release-activated calcium channel protein 1 (ORAI1) [66,67].

The oligomerization of STIM1/2 and ORAI1 proteins is the primary mechanism that restores ER Ca^2+^ levels, and recent insights into ER Ca^2+^ reduction show an association with ER stress [68,69]. Ca^2+^ homeostasis in the ER is essential because of Ca^2+^-dependent folding of newly synthesized proteins [70,71]; specifically, any decrease in the ER Ca^2+^ disrupts the function of GRP78/BiP and other chaperones, which causes the accumulation of unfolded proteins. Hence, when the ER transducers experience a lack of GRP78/BiP, they activate the transducers and promote the early or late response to unfolded proteins in the ER [72]. The importance of the various components of the SOCE machinery has been demonstrated in many disease conditions. In a rat model of ischemic stroke due to transient middle cerebral artery occlusion, STIM1/ORAI1 have shown to play a role in restoring neurological function [73]. This STIM1-ORAI1 interaction has also been shown to be functionally important in a cat model of hypertrophy-related calcium dysfunction in myocytes [74]. Similarly, Stim1 silencing by in vivo gene delivery of shRNAs, impairs the adaptive response to hypertrophy, which results in heart failure [75]. The critical effects of STIM1 and ORAI1 are seen in platelets during pathological thrombus formation [76,77] wherein STIM1 mediates the procoagulant activity and ORIA1 involves in platelet activation. Apart from STIM1, STIM2 has also been shown to be essential for regulating capacitive Ca^2+^ in neurons, apart from inducing hypoxic neuronal cell death [78]. Although the role of SOCE and its components (STIM1/2 and ORAI1) has been a major area of research focus in recent years, there is little or no evidence that links the SOCE machinery with ICH. Therefore, in the following sections, an attempt has been made to connect ER stress and cell death pathways in ICH.

## 6. Crosslinking ER Stress and ICH-Related Cell Death Pathways

**Figure 5:**
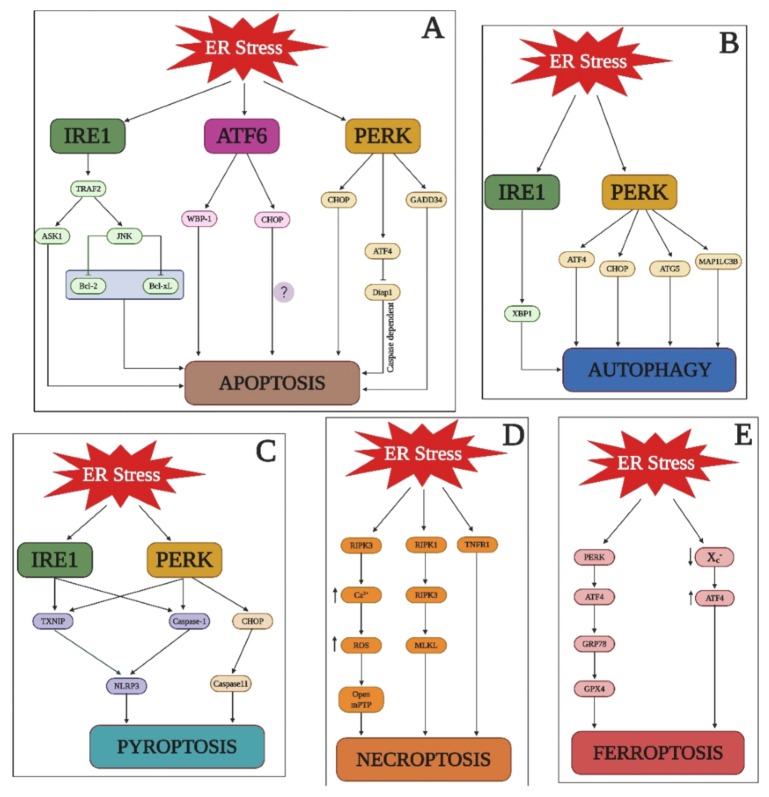
ER stress and cell death. A depiction of ER stress related signal transduction pathways that is responsible for apoptosis, autophagy, pyroptosis, necroptosis, and ferroptosis.

**Figure 6:**
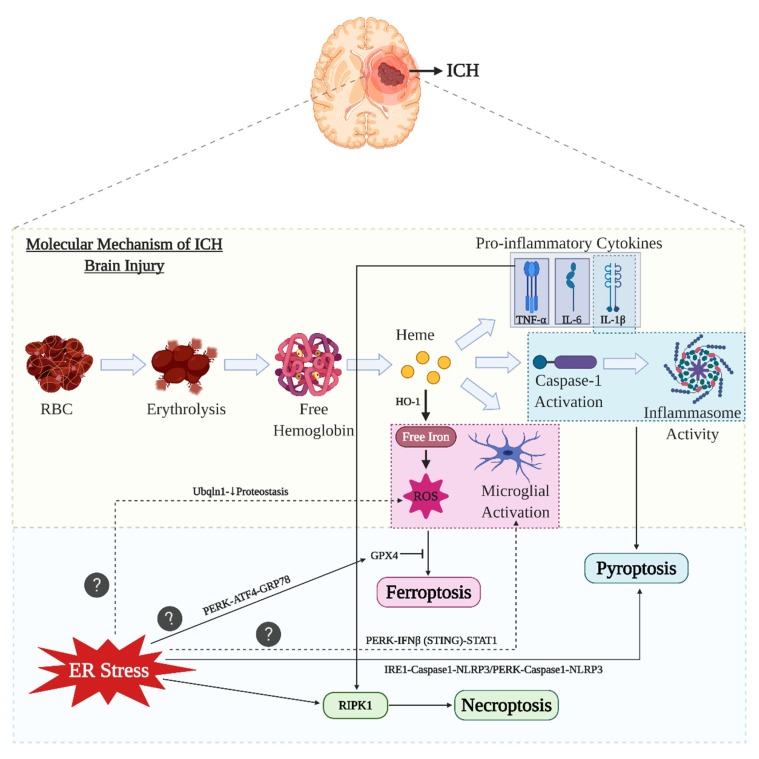
ER stress and ICH-related cell death pathways. The molecular mechanisms involved after ICH namely microglial activation and ROS production directly leads to ferroptosis by inhibiting GPX4. The role for ER stress to actively participate in ferroptosis is still a question. However, the role of ER stress is demonstrated to mediate GPX4 through its PERK arm. The caspase-1 activation and inflammasome activities after ICH leads to pyroptosis. The interrelation of the ER stress with pyroptosis involves both the IRE1 and PERK arms. The proinflammatory cytokine release responsible for neuroinflammation activates RIPK1 to further induce necroptosis. There is evidence that ER stress directly activating RIPK1 thereby causing necroptosis. However, the direct relationship with all these three ICH cell death pathways with that or ER stress is still not elucidated. However, future studies should be focused on these three unconventional ICH related cell death pathways wherein ER stress plays a key role. The role of proteostasis and PERK arm is also further explained to have a relationship with oxidative stress and microglial activation.

ICH disrupts cell metabolism and it is shown to activate a series of stress responses including ER stress [79]. On the other hand, ER dysfunction caused by protein misfolding and oxidative stress has led to acute central nervous system damage [80,81]. Recently, we have elucidated that acute phase of ICH has exacerbated the ER stress through proteasome overactivation, which also lead to neuroinflammation [82]. Although the studies to describe the relationship between ER stress and ICH are minimal, it is necessary to analyze the role of ER stress in ICH because of the crucial interplay between ER stress and various other factors involved in ICH pathophysiology including microglial activation, oxidative stress, neuroinflammation, and heme release [83,84,85]. An analysis of the function of ER stress components and their relationship with different ICH cell death pathways (apoptosis, autophagy, pyroptosis, necroptosis, and ferroptosis) will provide deeper insight into the critical contribution(s) of ER stress in post-ICH damage (Figure 5 and Figure 6).

### 6.1. ER Stress in Apoptosis

The ER transducers, IRE1, ATF6, and PERK, not only mediate ER stress but can also trigger proapoptotic signaling. This process occurs indirectly through downstream signaling molecules. The ER transducers are critical for ensuring homeostasis within a cell during ER stress and their effects are solely dependent on the intensity of the stress. For, e.g., in the context of prolonged ER stress, the prosurvival activity of the ER transducers switches to proapoptotic signaling, and PERK is responsible for attenuating the UPR [58]. The downstream translation of ATF4 from the PERK arm plays a role in both prosurvival and proapoptotic pathways during ER stress because it not only induces genes responsible for amino acid metabolism, protein secretion, and redox reactions, but also activates CHOP, which is responsible for promoting apoptotic cell death. On the other hand, under mild stress, PERK is important for ensuring pro-survival activities. However, the overexpression of other target genes, such as GADD34 from the PERK pathway, can also result in apoptosis.

An independent role for ATF4 in caspase-mediated apoptosis was demonstrated in a new drosophila model of chronic ER stress [53,61,86,87]. ATF6 has also been shown to induce CHOP but its relevance to the proapoptotic pathway is not well demonstrated; nonetheless, its relevance to apoptosis has been documented in myoblast cells wherein overexpression of ATF6 induced apoptosis by upregulating the WW domain binding protein (WBP)-1 [88]. Additionally, ATF6 plays a key role in apoptosis as the knockdown of ATF6 resulted in decreased apoptosis in mouse granulosa cells [89]. IRE1, on the other hand, has been shown to be involved in proapoptotic signaling and the activation of kinase pathways. During ER stress, IRE1 activates the c-Jun amino-terminal kinase (JNK) with the help of an adaptor protein called tumor necrosis factor receptor (TNFR)-associated factor-2 (TRAF2) [90]. This IRE1–TRAF2 complex in turn activates the apoptosis-signal-regulating kinase (ASK)-1 [91]; overactivation of ASK1 has been demonstrated during apoptosis of various cell types, especially neurons [92,93]. JNK, on the other hand, can also inhibit the anti-apoptotic activity of B-cell lymphoma (BCL)-2 and B-cell lymphoma-extra-large (Bcl-XL) by phosphorylating (BCL)-2 [94,95].

### 6.2. ER Stress in Autophagy

Autophagy is a prosurvival response to ER stress in many conditions and is essential for removing misfolded proteins [96]. The induction of autophagy by ER stress has been shown to be both beneficial and detrimental for the cells. When cells are exposed to hypoxic conditions, there is an increase in ER stress, which induces autophagy in an ATF4-dependent manner, thus extending our understanding into the mechanistic link between the UPR and autophagy [97]. The relationship between PERK and autophagy has been described in human cancer cells lines wherein hypoxia increased the transcription of genes that are important for autophagy, namely microtubule-associated protein 1 light chain 3β (MAP1LC3B) and autophagy-related gene 5 (ATG5); the latter represents the association between the PERK arm of the UPR system after ER stress and hypoxia-induced autophagy [98]. The downstream regulators of the PERK arm (ATF4 and CHOP) have been shown to increase the transcription of autophagy genes [99]. UPR branches and their downstream proteins play a major role in autophagy but they may either induce or inhibit it as the proteins downstream of IRE1 (XBP1) promote both induction and inhibition of autophagy. Specifically, the PERK downstream protein (ATF4) and the ATF6 arm of the UPR induce autophagy [100].

### 6.3. ER Stress in Pyroptosis

Recent research has demonstrated a relationship between ER stress and inflammasome activation in various pathologies. One study in a rat model of spinal cord injury has described that the thioredoxin interacting protein (TXNIP)—a protein induced by ER stress—activates NLRP3 inflammasomes. However, that study did not clarify the relevant molecular pathways between inflammasome and ER stress [101], but earlier studies have shown the importance of TXNIP in inflammasome pathways during ER stress [102,103]. Pyroptosis occurred due to the involvement of TXNIP with PERK and IRE1α, which induced IL-1β mRNA transcription and IL-1β production with the help of the NLRP3 inflammasome [104]. Another study in a hepatocyte model demonstrated that ER stress led to NLRP3 inflammasome activation by PERK and IRE1α, which resulted in the overexpression of caspase-1 and led to pyroptosis [105]. In an ischemia-reperfusion-induced acute kidney injury model, ER stress activated the CHOP-caspase-11 pathway, thereby inducing pyroptosis of the renal tubule epithelial cells [106]. A role for ER stress in the activation of inflammasomes and IL-1β production has been reported to be pivotal in liver damage and steatosis [107].

### 6.4. ER Stress in Necroptosis

ER-stress-induced necroptosis has been shown by multiple studies. In a mouse model of cardiac ischemia-reperfusion (IR) injury, the RIPK family of proteins (RIPK3) stimulated ER stress and mediated necroptosis. The underlying signal transduction pathway consisted of an increase in calcium in the ER following an increase in ROS; this opened the mitochondrial permeability transition pore (mPTP), causing necroptosis of the cardiomyocytes. Additionally, overexpression of ER stress markers (GRP78, CHOP, and PERK) was observed in the cardiomyocytes of IR mice compared to RIPK3 knockout mice [108]. Another study aimed to elucidate the role of ER stress in inducing necroptosis through the RIPK1/RIPK3/mixed lineage kinase domain-like protein (MLKL)-dependent pathway in L929 cells and demonstrated that necroptosis was indeed TNFR1 dependent [109].

### 6.5. ER Stress in Ferroptosis

A possible relationship between ER stress and ferroptosis was unraveled in a glioma model, wherein the PERK-ATF4-GRP78 pathway inhibits GPX4, which in turn modulates ferroptosis. Interestingly, the inhibition of ER stress enhanced ferroptosis and complemented the anticancer activity of dihydroartemisinin [110]. Although a direct relationship between ER stress and ferroptosis has not yet been established, it is known that the inhibition of cystine/glutamate transporter (xc−) increases ER stress by upregulating ATF4, and that it also increases ferroptosis in cancer cell lines [111].

## 7. ER Stress Components and ICH Therapeutic Strategies

By understanding the relationship between ER stress in either activation of inhibition of other prominent factors of ICH including microglial activation [5,9], ROS production [6], proinflammatory cytokine release [11], and heme and iron release [13,14]; we will have a greater knowledge on the molecular mechanism involved in ICH induced secondary brain injury. In a traumatic brain injury (TBI) model, increased PERK phosphorylation resulted in the excessive release of interferon (IFN)-β through stimulator of interferon gene (STING) signaling pathway. IFN-β, in turn, causes microglial activation via STAT1 signaling pathway and the release of proinflammatory cytokines. The study also demonstrated that blocking PERK-phosphorylation by either GSK2656157 or PERK knockdown prevents the activation of microglia [112].

Apart from microglial activation, another important factor in ICH-induced neuroinflammation is oxidative stress. The interrelation between proteostasis and oxidative stress was demonstrated in the ischemic stroke mouse model: administration of L-2-oxothiazolidine-4-carboxylic acid (OTC), a precursor of cysteine, upregulates ubiquilin-1 (Ubqln1) and enhances proteostasis and glutathione (GSH) level. GSH is an important antioxidant synthesized in cells, which detoxifies ROS. However, the therapeutic effect of OTC diminishes in Ubqln1 knockout mice after cerebral stroke, suggesting that the therapeutic potential of OTC involves both antioxidative property and proteostasis enhancement [113]. Another study from the same group also demonstrated that the administration of nialamide (a non-selective monoamine oxidase inhibitor) in the ischemic stroke mouse model can also upregulate Ubqln1, and in turn reduces oxidative stress as well as neuroinflammation (Figure 6) [114].

The activities of the three ER transducers in promoting UPR represent an ideal target for mitigating ER stress, but if the sensors are activated for prolonged periods of time, the UPR mechanism turns detrimental in all cell types, including neuronal cells. Protein translation overload is temporarily halted by PERK; hence, the function of PERK is extremely important for easing the UPR. PERK is functionally essential for deciding neuronal fate because it is a crucial mediator of ER stress [115], and numerous pharmacological agents are capable of targeting the UPR and ER stress components. Thus, existing approaches to treat ICH can be combined with pharmacological agents that can directly activate or deactivate ER stress components, which might play a critical role in alleviating post-ICH symptoms.

The PERK/eIF2α pathway and its downstream regulators have been shown to be upregulated in studies related to cerebral ischemia [116], recurrent febrile seizures [117], and traumatic brain injury [118], and the involvement of PERK has been previously documented in a brain ischemia and reperfusion model, with the help of eIF2α. [119]. In an autologous blood infusion model of ICH, PERK was upregulated with the help of phosphorylated-eIF2α and ATF4. Due to RBC lysis and free hemoglobin release, heme exposure is prevalent in the perihematomal area after ICH [7]. The PERK arm of the ER stress sensors is activated within 3 h after its exposure to heme in human aortic smooth muscle cells, which might lead to their apoptosis [120].

It is important to note that there are few recent studies that explain the direct relationship between ER stress and ICH with specific inhibitors targeting ER transducers. In one study, the secondary brain injury post ICH was shown to increase the protein levels of p-eIF2α and ATF4 ultimately leading to proapoptotic neuronal cell death. Using a PERK antagonist (GSK2606414) and a selective inhibitor of eIF2α dephosphorylation, it was shown that neuronal apoptosis is reduced following ICH. PERK inhibition by GSK260414 also resulted in the decrease of CHOP and cleaved caspase-12. Although the study relates PERK signaling pathway inhibition with ICH, the authors have emphasized to have a closer look into the role of PERK and its interaction with the enzymatic activity of calcineurin. As calcium overload in the cytoplasm is also said to be a possible factor for apoptosis, which is induced by calcineurin. By understanding the interrelationship between calcineurin and PERK signaling pathway we shall have a clear insight into the mechanism of neuronal apoptosis post-ICH [121].

Post-ICH, SBI events overrun the inflammatory response, leading to neuroinflammation. This process might significantly increase the activity of other ER stress sensors. The neuroprotective effect of DiDang Tang (DDT; a Chinese traditional medical formula) was clarified in neuronal cells and rats. DDT was shown to mediate through blocking of GRP78-IRE1/PERK pathway. The study confirms that DDT exerted a neuroprotective effect when it is pretreated in the neuronal cells and the pretreated cells showed less ER stress, Ca^2+^ overload and mitochondrial apoptosis. In addition to the role of PERK and ATF6 involvement in ICH as stated in the aforementioned two studies, this study clarified the role of the inhibition of evolutionarily conserved ER stress sensor, IRE1, and its neuroprotective effect in rats and the neuronal cell model of ICH [122]. One other study explained the role of ATF6 in ICH. Post-ICH, there is an increase in the expression of ATF6 and suppression of ATF6 with melatonin is shown to decrease the brain injury and enhance the neurological functions. The study also explained the decrease in apoptotic neuronal cells were through ATF6 inhibition, which resulted in the reduction of CHOP, Bcl-2-associated X protein (Bax), and cleaved caspase-3. Furthermore, inhibition of CHOP signaling by siRNA has been shown the proapoptotic nature of ATF6 [123].

## 8. Conclusions

Although the pathophysiological symptoms of ICH are known to begin with PBI, SBI plays an important role in the injury mechanism. Various stresses have been shown to contribute to SBI, and all stress factors, from oxidative stress to ER stress, directly or indirectly increase the severity of SBI. ER stress, in combination with the oxidative stress and other potential causes of damage in the injury site, including resident microglia and infiltrating macrophages, contribute to a delay in the healing process. We believe that narrowing down possible therapeutic targets by analyzing each and every factor with evidence-based research is the only way to arrive at a precise therapeutic approach. In this review, we tried to elucidate the pivotal role of ER stress after SBI by considering various cell death pathways that can be induced after prolonged ER stress in ICH. Furthermore, elucidating a role for ER transducers, which may be potential effectors of various cell death pathways in ICH, apart from the traditional process of autophagy, apoptosis, and necrosis, represent added information in this review.

As far as ICH is concerned, research has started to shed light on the concept of cell death mechanisms and possible components that might serve to restore homeostasis; of these, ER stress is crucial. Future studies should address the downstream aspects of ER stress components and their implications in both PBI and SBI.
